# Supplementary material for: Encoding of odor information and reward anticipation in anterior cortical amygdaloid nucleus
Source: iScience. 2026 Jan 7;29(2):114590. doi: 10.1016/j.isci.2025.114590 (PMC12874591; doi:10.1016/j.isci.2025.114590)
Supplement: Document S1. Figures S1–S4 [file mmc1.pdf]

iScience, Volume ■ ■

Supplemental information

Encoding of odor information and reward anticipation in anterior cortical amygdaloid nucleus

Kazuki Shiotani, Yuta Tanisumi, Junya Hirokawa, Yoshio Sakurai, and Hiroyuki Manabe

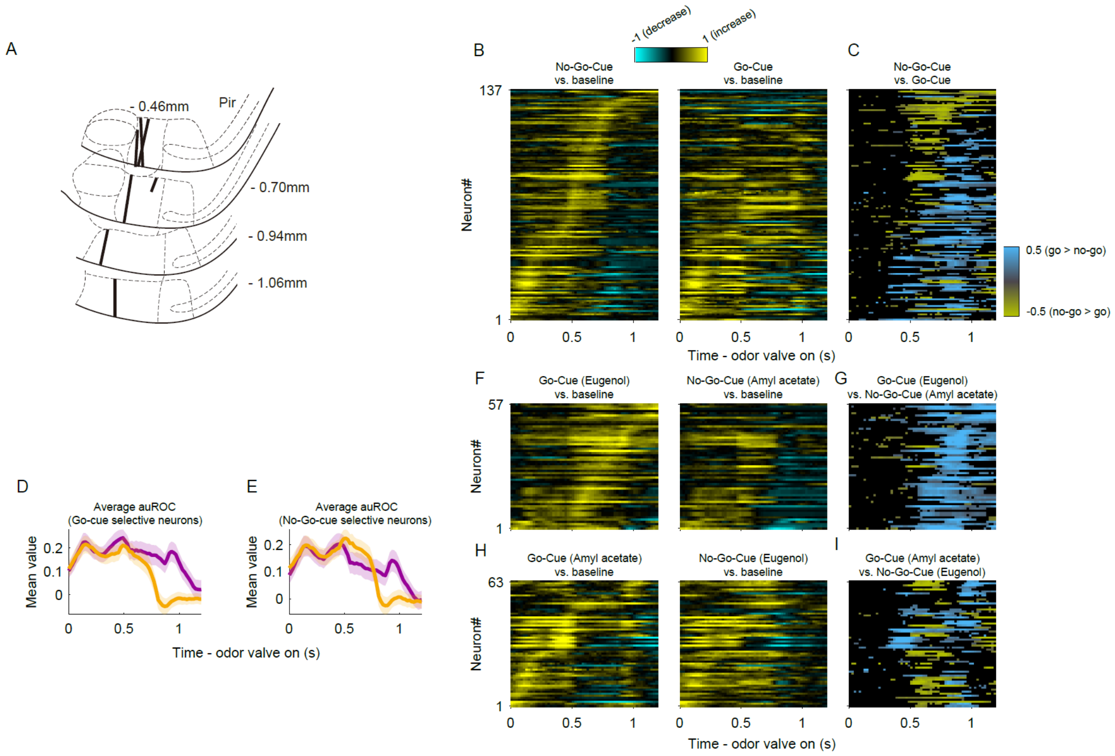

Supplementary Figure S1. auROC analysis of go- and no-go-cue selective neurons in the ACo, related to Figure 1.

(A) Recording tracks (vertical thick lines) of the ACo. Pir, piriform cortex.
(B) Normalized firing rates (auROC values) for no-go-cue selective neurons (n = 137). auROC values (aligned by odor valve opening) were calculated by go-cue odor presentation versus baseline (left) and no-go-cue odor presentation versus baseline (right) in the sliding bins (width, 100 ms; step, 20 ms). Yelllow, increase from baseline; blue, decrease from baseline. Each row corresponds to one neuron, with neurons in the left and right graphs in the same order. Neurons are sorted by the peak time for auROC values calculated by go-cue odor presentation versus baseline.
(C) Cue preference curves (auROC values, go-cue versus no-go-cue odor presentation, aligned by odor valve opening, odor port exit) for no-go-cue selective neurons. Each row corresponds to one neuron, with neurons in the left and right graphs in the same order of (B). Color scale indicates significant preferences ($p < 0.01$, permutation test; positive values correspond to the go-cue preferred responses). The black boxes indicate bins with non-significant preferences ($p > 0.01$, permutation test).
(D) Mean auROC values of go-cue selective neurons. Shaded areas indicate SEM. auROC values (aligned to odor valve opening) were calculated for go-cue odor presentation versus baseline, and for no-go-cue odor presentation versus baseline.
(E) Same as (D), but for no-go-cue selective neurons.
(F) Same as (B), but for go-cue selective neurons with eugenol as the go odor and amyl acetate as the no-go odor (n = 57).
(G) Same as (C), but for go-cue selective neurons with eugenol as the go odor and amyl acetate as the no-go odor.
(H) Same as (B), but for go-cue selective neurons with amyl acetate as the go odor and eugenol as the no-go odor (n = 63).
(I) Same as (C), but for go-cue selective neurons with amyl acetate as the go odor and eugenol as the no-go odor.

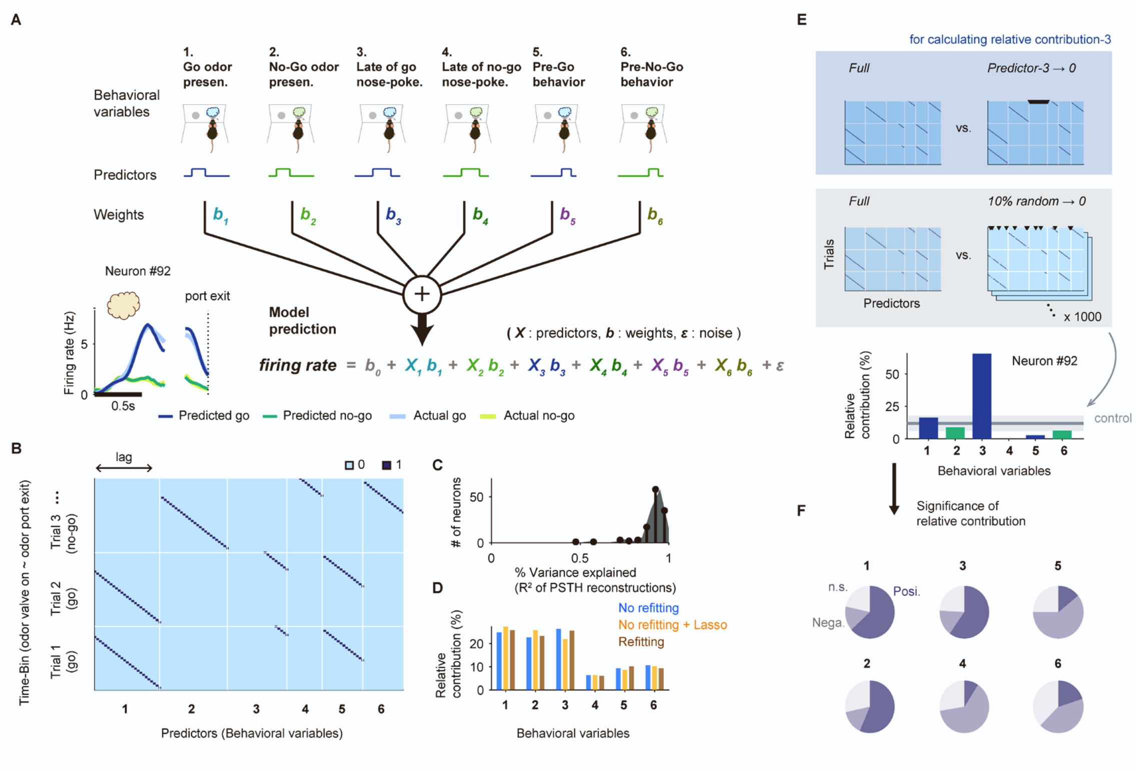

Supplementary Figure S2. Generalized linear models and the relative contributions of behavioral variables to neural activity, related to Figure 2.

(A) Schematic of the encoding model used to quantify the relationship between behavioral variables and the activity of each neuron. Inset, predicted and actual averaged firing rate relative to the odor onset and odor port exit for one neuron.

(B) Structure of predictor matrices. The predictor has columns for each variable, which take non-zero values for time bins (rows) corresponding to the appropriate time offset from the given event.

(C) Variance explained (R^2 of PSTH reconstructions) between predicted and actual averaged firing rate relative to the odor onset and odor port exit across the go-cue-selective neurons.

(D) Average relative contributions across the go-cue-selective neurons assessed separately using three different approaches: no refitting (used in the paper); no refitting + Lasso regularization; and refitting. Lasso regularization was applied using the lasso function in MATLAB; the mean square error (MSE) of the model was estimated using fivefold cross-validation, and we chose the lambda value that minimized the MSE. The results with lasso regularization were almost identical to the result without regularization, which suggests that there was no significant overfitting in our model.

(E) Evaluation for significance of relative contributions assessed no refitting approach. The partial model was equivalent to the full model, except that the randomly selected β values of the predictors of the excluded variable (10% of predictors) were set to zero, in which processing was performed 1,000 times. Using the control mean ± 2 standard deviation (SD), the statistical significance was determined (< mean – 2SD, negative relative contribution; > mean + 2SD, positive relative contribution).

(F) Proportions of the significance of relative contributions for each behavioral variable across the go-cue-selective neurons.

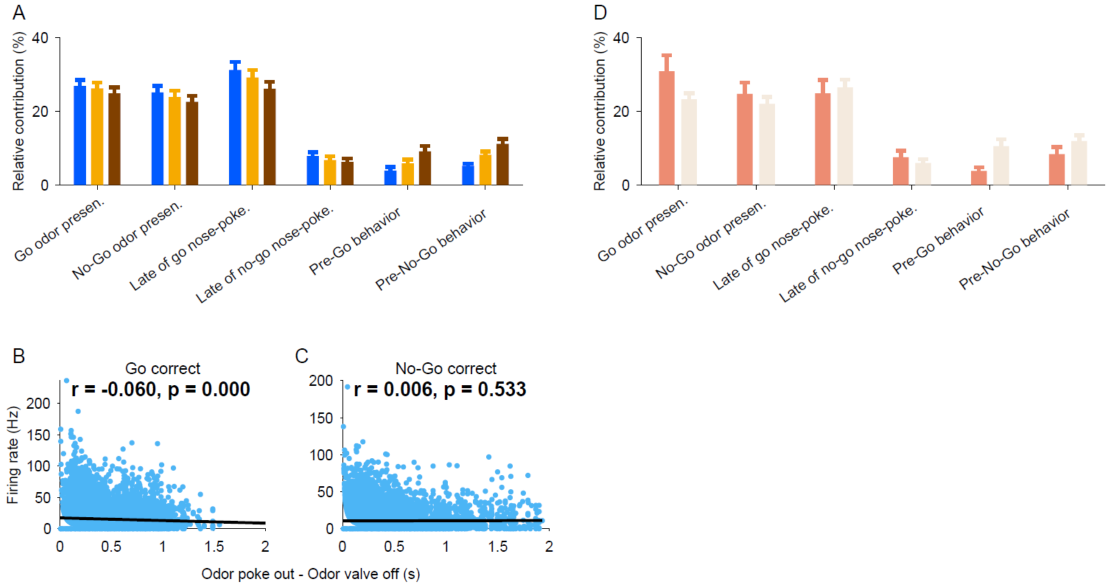

Supplementary Figure S3. Behavioral variable contributions and trial progression effects in go-cue-selective neurons, related to Figure 2.

(A) Relative contribution of each behavioral variable to the explained variance of the neural activity, averaged across the go-cue-selective neurons, when the pre-exit timing was set to 100, 200, or 300 ms. All error bars represent the standard error of the mean. Blue, yellow, and brown bar represent 100 ms, 200 ms, and 300 ms, respectively. There were no significant differences in each behavioral variable between the three conditions (Tukey's test, $p>0.05$).

(B) Duration from odor poke out to odor valve off and the firing rate of go-cue-selective neurons during that period in go correct trials. Each dot represents a trial.

(C) Same as (B), but for no-go correct trials.

(D) Relative contribution of each behavioral variable to the explained variance of the neural activity, separated into those whose go-cue responses showed a significant correlation with trial progression and those that did not. All error bars represent the standard error of the mean.

Red bar indicates neurons whose go-cue responses showed a significant correlation with trial progression, whereas cream bar indicates those without such correlation. There were no significant differences in each behavioral variable between the two conditions (paired ttest, $p>0.05$).

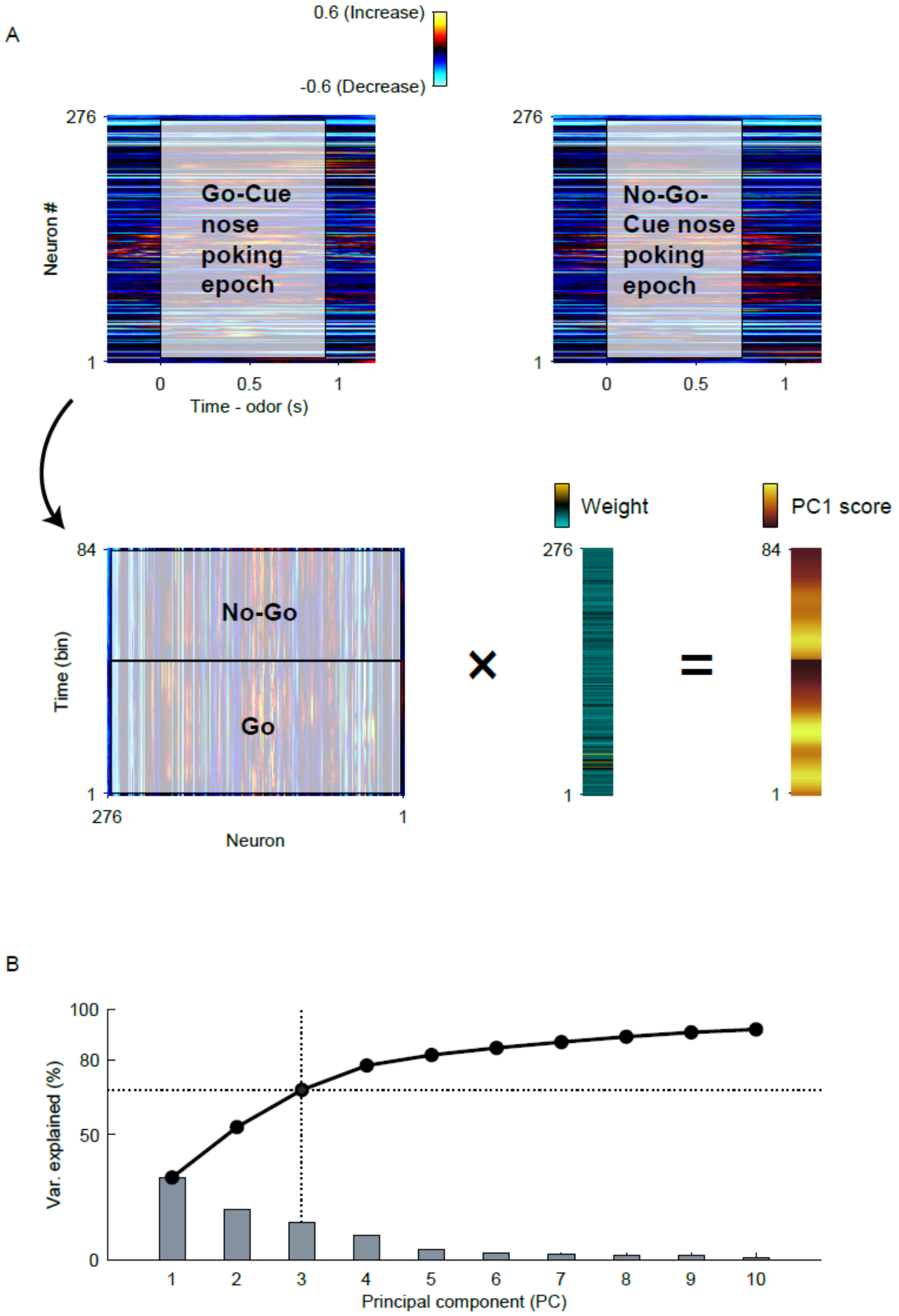

Supplementary Figure S4. Population vector construction and analysis for ACo neuron population response, related to Figure 3.

(A) Population vector construction. We constructed the two conditions (91 time bins) × 276 neurons matrix during the nose-poking epoch, within which, the columns contained the auROC values corresponding to the trial-averaged firing rate changes from the baseline. By performing principal component analysis (PCA) on the dataset, we reduced the dimensionality of the ACo population from 276 neurons to three principal components (PCs). Subsequently, we obtained the nose-poking epoch subspaces (graphs show the values of the first dimension of the nose-poking epoch subspaces).

(B) Screen plot of the nose-poking epoch subspaces. It is notable that we used the three subspaces because they explained 67.9% of the total variance.
